# Long-Term Intravitreal Ranibizumab as a Potential Additional Risk Factor for Neurodegeneration in Parkinson’s Disease: A Case Report

**DOI:** 10.3389/fphar.2018.00608

**Published:** 2018-06-08

**Authors:** Gianluca Trifirò, Ilaria Marcianò, Paola M. Cutroneo, Edoardo Spina, Eliana Mirabelli, Costantino J. Trombetta, Francesca Morgante

**Affiliations:** ^1^Department of Biomedical and Dental Sciences and Morpho-functional Imaging, University of Messina, Messina, Italy; ^2^Unit of Clinical Pharmacology, Azienda Ospedaliera Universitaria Policlinico “G. Martino” – Messina, Italy; ^3^Department of Clinical and Experimental Medicine, University of Messina, Messina, Italy; ^4^Neurosciences Research Centre, Molecular and Clinical Sciences Research Institute, St George’s University of London, London, United Kingdom

**Keywords:** anti-vascular endothelial growth factors, ranibizumab, Parkinson’s disease, intravitreal, neurodegeneration

## Abstract

In November 2012, a 72-year old patient was diagnosed with left eye wet age-related macular degeneration. The patient received three monthly intravitreal injections of ranibizumab, with complete resolution of retinal hemorrhage and edema and reinstatement of visual acuity. In May 2015, symptomatic relapse was detected. The patient was again treated with intravitreal ranibizumab, with overall six injections till the end of February 2016. In May 2016, the patient complained of left hand resting tremor, bradykinesia, and postural rigidity of head and trunk. A diagnosis of clinically established PD was made based on new criteria of the Movement Disorders Society. Single Photon Emission Computerized Tomography of the Dopamine Transporter with (123I) ioflupane documented a low Dopamine Transporter (DAT) uptake mostly in the right striatum. Due to the documented protective role of vascular endothelial growth factor (VEGF) on the dopaminergic neurons, intensive intravitreal injections of the anti-VEGF agent ranibizumab may have played as an additional risk factor accelerating the neurodegeneration process related to PD and the onset of the related clinical signs and symptoms.

## Introduction

Age-related macular degeneration (AMD) is a chronic progressive disorder of the retina, representing the main cause of irreversible vision loss in elderly people (de Jong, 2006) in both Western and Eastern Countries.

The neovascular or “wet” AMD is characterized by choroidal neovascularization and vascular leakage, associated with up-regulation of angiogenic factors, such as vascular endothelial growth factor (VEGF), a cytokine that promotes vascular permeability and angiogenesis. Through reduction of edema and new blood vessel growth, VEGF inhibitors administered by intravitreal injection are an established treatment for “wet” AMD and include bevacizumab (only as off-label use), pegaptanib, and, more recently, ranibizumab and aflibercept.

Recent studies revealed that VEGF displays a key role in neuronal survival and proliferation (Drake and Little, 1995; Silverman et al., 1999; Storkebaum and Carmeliet, 2004), with neuroprotective effects, due to apoptosis inhibition, stimulation of neurogenesis, and activation of antioxidants (Zachary, 2005; Krum et al., 2008; Nowacka and Obuchowicz, 2012; Quittet et al., 2015). This effect has been highlighted also on dopaminergic (DA) neurons and on both *in vitro* and *in vivo* pre-clinical studies on Parkinson’s disease (PD) models (Yasuhara et al., 2004, 2005a,b; Yasuda et al., 2007; Falk et al., 2011; Piltonen et al., 2011; Yue et al., 2014). Inhibition of the neuroprotective effects associated to VEGF may theoretically play a role in the development of neurodegenerative disorders involving dopamine transmission, including PD.

We report a case of PD which occurred after long-term treatment with intravitreal injections of ranibizumab for the treatment of wet AMD.

## Case Report

In November 2012, a 72-year old man was diagnosed with wet AMD in his left eye, based on fundus examination and optical coherence tomography (OCT), which was requested for the onset of metamorphopsia. At that time, he was treated with combination of angiotensin converting enzyme inhibitor plus thiazide diuretic for a 20-year history of well controlled hypertension.

His best-corrected visual acuity in the right and left eyes was 10/10 and 8/10, respectively. On slit-lamp examination, both anterior chambers showed clear aqueous humor and no inflammatory reaction. Dilated fundus examination revealed a subretinal whitish mass and adjacent subretinal hemorrhage. OCT confirmed the presence of a subretinal lesion and intraretinal edema. After obtaining informed consent, the patient was monthly treated with intravitreal administration of 0.5 mg ranibizumab for three months, without any complication and with complete retinal hemorrhage and edema resolution and increased visual acuity of left eye (10/10). Thereafter, the patient underwent routine follow-up visits, on a 2-month basis, including fundus examination and OCT which did not document any abnormal finding. In May 2014, a reduction of visual acuity (from 10/10 to 7/10) was registered. The patient was periodically followed-up but not treated with anti-VEGF drugs as there was no sign of neovascularization. In May 2015, visual acuity further reduced to 3/10 and both fundus examination and OCT revealed a reactivation of the neovascular membrane, edema and pigment epithelial detachment. For this reason, the patient was again treated with intravitreal injections of ranibizumab (0.5 mg), firstly on a monthly basis and thereafter using *treat and extend* approach, with overall six injections till the end of February 2016, when visual acuity increased to 6/10. At the follow-up visit in May 2016, the neovascular membrane appeared inactive and the visual acuity was stable at 6/10, so the ophthalmologist decided for a *pro re nata* approach (i.e., as needed).

In the same period, the patient referred to the Movement Disorders Clinic due to intermittent tremor on the left hand, started around February 2016. He did not complain non-motor symptoms.

Neurological examination disclosed resting tremor on the left hand, mild bradykinesia of left lower limb, and mild rigidity of head and trunk. His motor Unified Parkinson’s Disease Rating Scale (UPDRS) was 11/108.

The patient had no family history of PD or other neurodegenerative diseases nor had he been ever exposed to pesticides. Magnetic Resonance Imaging of the brain showed rare small subcortical white matters hyperintensities on T2 (mainly periventricular and frontal) and some bilateral hypointensities in T1 in the striatum, more prominent on the right, compatible with small ischemic lesions.

**FIGURE 1:**
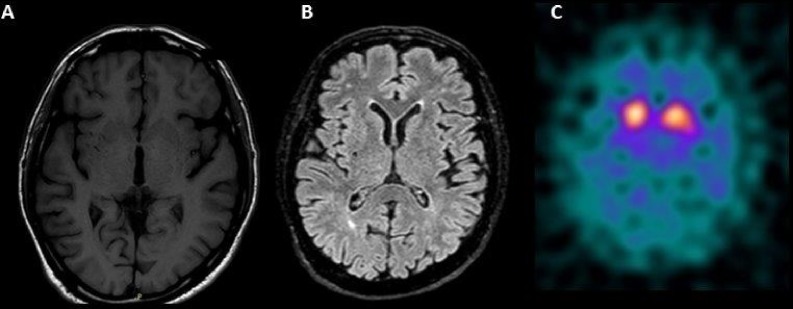
**(A)** Bilateral hypointensities in T1 in the striatum, more prominent on the right side and **(B)** rare small subcortical white matters hyperintensities on T2 (mainly periventricular and frontal) on Magnetic Resonance Imaging of the brain. **(C)** Single Photon Emission Computerized Tomography of the Dopamine Transporter (DAT) with 123I-ioflupane showing a significant low uptake of DAT, mostly in the right striatum.

Single Photon Emission Computerized Tomography (SPECT) of the Dopamine Transporter (DAT) with 123I-ioflupane documented a significant and clear low uptake of DAT, mostly in the right striatum (**Figure 1**). A diagnosis of clinically established PD was made based on new criteria of the Movement Disorders Society (Postuma et al., 2015).

A treatment with levodopa/carbidopa (300 mg/daily) was started at the beginning of 2017, due to worsening of tremor and bradykinesia leading to gait impairment and fatigue (motor UPDRS = 15/108). At follow-up examination in May 2017, response to levodopa was demonstrated by improvement of motor symptoms (motor UPDRS = 6/108), particularly of gait. Up to November 2017 other two injections of ranibizumab were intravitreally administered with visual acuity equal to 3/10 and the patient was in stable treatment with levodopa/carbidopa (300 mg/daily).

## Discussion

We described the development of PD in a patient without any genetic and environmental risk factor for PD, who received repeated intravitreal injections of ranibizumab for wet AMD over the previous year. The clinical picture with lack of atypical features is consistent with the clinical diagnosis of PD. The DAT SPECT evidence of pre-synaptic dopamine loss supports the presence of neurodegeneration in the nigro-striatal pathway, typical of neurodegenerative Parkinsonism. Moreover, brain MRI did not disclose areas of cortical and subcortical atrophy, suggesting the diagnosis of atypical Parkinsonism. The presence of vascular lesions in the basal ganglia at brain MRI does not exclude the diagnosis of idiopathic PD, as they have been described in PD patients with vascular risk factors, such as hypertension (Antonini et al., 2012). On the other hand, there is a strong biological plausibility and temporal relationship between intensive intravitreal administration of ranibizumab and onset of PD-related signs and symptoms which may suggest a role of the anti-VEGF drug as additional risk factor resulting potentially in accelerating the PD-related neurodegeneration process.

PD is a multi-systemic neurodegenerative disease, primarily affecting DA neurons of substantia nigra, and characterized by intracellular accumulation of the protein α-synuclein (Poewe et al., 2017). Mechanisms of neurodegeneration hypothesized in PD include abnormal α-synuclein degradation by the ubiquitin–proteasome system and the lysosomal autophagy system, propagation of α-synuclein with a prion-like mechanism, mitochondrial dysfunction, oxidative stress, and neuroinflammation (Poewe et al., 2017). Recent studies demonstrated in PD patients the presence of α-synuclein in the retina (Bodis-Wollner et al., 2014) as well the presence of specific OCT abnormalities, such as reduced parafoveal thickness (Slotnick et al., 2015). The retina has several molecular and cellular features in common with the brain, such as neurons, glial cells, connected vasculature, and a blood barrier (Trost et al., 2016). The most liable mechanisms potentially explaining the relationship between AMD and PD are related to neurodegenerative processes and chronic inflammation: the activation of microglia cells in the retina and nervous system may trigger the inflammatory response and aggravate both the retinal and DA degeneration (Whitton, 2007). Ranibizumab is a humanized recombinant monoclonal antibody fragment targeted against human VEGF. It binds with high affinity to the VEGF-A isoforms, thus preventing binding of VEGF-A to its receptors (European Medicines Agency. Lucentis – Summary of Product Characteristics. Available from: http://www.ema.europa.eu/docs/en_GB/document_library/EPAR_-_Product_Information/human/000715/WC500043546.pdf. Accessed 01 Feb 2018). Ranibizumab maintains effective retinal concentrations for around 1 month, being able to reach low concentrations in the serum compartment as well (Stewart, 2012; Gibson and Gibson, 2014). Based on the Summary of Product Characteristics (European Medicines Agency. Lucentis – Summary of Product Characteristics. Available from: http://www.ema.europa.eu/docs/en_GB/document_library/EPAR_-_Product_Information/human/000715/WC500043546.pdf. Accessed 01 Feb 2018), following monthly intravitreal injections in AMD patients, serum concentrations of ranibizumab generally range between 0.79 and 2.90 ng/ml. Such concentrations are below the drug concentration necessary to inhibit the VEGF activity by 50% (IC_50_ = 11–27 ng/ml). Serum ranibizumab concentrations are predicted to be approximately 90,000-fold lower than vitreal ranibizumab concentrations. Results from clinical trials documented no significant reduction in serum VEGF levels within the first 1 month of treatment (Zehetner et al., 2015), but a significant decline in systemic VEGF levels was detected after 1 and 2 years (Enders et al., 2015). The resulting systemic VEGF inhibition after intravitreal injections may lead to systemic effects (Gibson and Gibson, 2014). Clinical trials on ranibizumab showed a high incidence of stroke, myocardial infarction and non-ocular hemorrhage (Brown et al., 2006; Rosenfeld et al., 2006), further confirmed by recent observational studies (Kemp et al., 2013; Pratt et al., 2014). Furthermore, a meta-analysis of randomized trials on ranibizumab for AMD treatment identified a stronger relationship of systemic vascular adverse events and intravitreal administration of VEGF inhibitors in case of more intensive treatment schedule (Ueta et al., 2014).

Recent evidence suggested that the VEGF signal pathway may directly and indirectly improve DA neuron survival (Silverman et al., 1999; Pitzer et al., 2003; Yasuhara et al., 2004). VEGF administration inhibits DA neurons loss in PD models, especially in the substantia nigra and in the striatum (Sheikh et al., 2017), thus representing a potential therapeutic target for prevention of DA neuron death and PD progression. Functional polymorphisms of the VEGF gene expression have been associated with an increased risk of developing PD in the Chinese Ham population (Wu et al., 2016). It has been postulated that reduced VEGF levels may cause neurodegeneration, by impairing neural tissue perfusion, thus causing ischemia and production of free radicals (Storkebaum and Carmeliet, 2004). This hypothesis is consistent with a recent autopsy study in patients with dementia with Lewy body, in whom VEGF deficiency has been associated with a loss of microvessels and low occipital blood flow (Miners et al., 2014). On the other hand, VEGF is up-regulated by ischemic and inflammatory stimulation, which accompany neurodegeneration. The state of brain hypoxia up-regulates VEGF and its subsequent higher levels mediate angiogenesis, causing microvascular leakage and fragility. These changes lead to edema, bleeding, and impair neural tissue perfusion. The resultant hypoxia then further up-regulate VEGF levels again (Storkebaum and Carmeliet, 2004).

VEGF shows neuroprotective effects, due to apoptosis inhibition, stimulation of neurogenesis, and activation of antioxidants (Zachary, 2005; Krum et al., 2008; Nowacka and Obuchowicz, 2012; Quittet et al., 2015). Specifically, ranibizumab binds to the VEGF-A, one of the five isoforms of the VEGF family and one of the strongest inducers of vascular permeability (Yla-Herttuala et al., 2007), which showed neuroprotective effects in several *in vitro* and *in vivo* PD models (Silverman et al., 1999; Pitzer et al., 2003; Yasuhara et al., 2004; Tian et al., 2007; Yue et al., 2014). A recent study on the Italian ADR Spontaneous Reporting System showed 3 reports of “extrapyramidal syndrome” due to intravitreal bevacizumab and 1 to intravitreal aflibercept (Cutroneo et al., 2017). Similarly, in the Food and Drug Administration’s Adverse Event Reporting System, three cases of neurodegenerative disorders have been reported following bevacizumab use (Shamloo et al., 2012). On the other hand, the onset of such disorders may be due to vascular events and/or stroke, which are well known and listed risks of intravitreal therapy with anti-VEGF drugs in general, including ranibizumab. Finally, a meta-analysis of randomized trials on ranibizumab for AMD treatment identified a stronger relationship of systemic vascular adverse events and intravitreal administration of VEGF inhibitors in case of more intensive treatment schedule (Ueta et al., 2014).

All the above described experimental and epidemiological evidence raise the hypothesis that persistent and intensive VEGF inhibition concurred to DA degeneration in our patient. AMD may be considered as a neurodegeneration of the retina (de Jong, 2006), thus potentially sharing with PD common biological pathways, such as oxidative stress and inflammation (Ding et al., 2009; Poewe et al., 2017). Chung et al. (2014) investigated the risk of developing PD within 3 years after the AMD diagnosis in a retrospective cohort, population-based, claims database study in Taiwan . Adjusting for several vascular risk factors (i.e., coronary heart disease, hypertension, diabetes, and hyperlipidemia), subjects with AMD had a significant higher risk of developing PD than AMD-free patients during follow-up. On the contrary, a case-control study found no statistically significant differences in the frequency of AMD between patients with idiopathic PD and healthy subjects (Nowacka et al., 2014). Using claims databases from the United States, Brilliant et al. (2016) found that around 70% of the levodopa users started the drug treatment after AMD diagnosis . However, the use of anti-VEGF drugs, which are on the market since 2005, was not considered in any of the studies and it cannot be excluded that anti-VEGF drugs rather than AMD itself triggered the clinical manifestation of PD.

## Conclusion

In conclusion, our case report points out to VEGF inhibition as a possible additional risk factor of neurodegeneration of DA neurons in PD.

According to the Naranjo causality assessment scale (Naranjo et al., 1981), the causal relationship between use of ranibizumab and PD development was scored as “possible”.

In particular, we hypothesize that long-term treatment with intravitreal ranibizumab led to a persistent inhibition of VEGF activity which played an important compensatory neuroprotective role in older patient with AMD, ultimately triggering PD.

## Ethics Statement

A signed statement of informed consent to publish the case description was obtained from the patient.

## Author Contributions

EM, CT, and FM collected the data and performed the investigation. GT, IM, PC wrote the manuscript in collaboration. ES and FM critically reviewed the manuscript for important intellectual content and suggested valuable comments, which improved the quality of the manuscript.

## Conflict of Interest Statement

FM declares she receives royalties from publication of the book “Disorders of Movement” (Springer 2016). She has been part of advisory boards of Medtronic, Merz, and UCB Pharma. She has received honoraria for speaking from UCB Pharma, Medtronic, Chiesi, Abbvie, Allergan, Merz, and Zambon. The other authors declare that the research was conducted in the absence of any commercial or financial relationships that could be construed as a potential conflict of interest.
